# Chemopreventive and immunomodulatory effects of *Murraya koenigii* aqueous extract on 4T1 breast cancer cell-challenged mice

**DOI:** 10.1186/s12906-015-0832-z

**Published:** 2015-09-04

**Authors:** Swee Keong Yeap, Nadiah Abu, Nurul Elyani Mohamad, Boon Kee Beh, Wan Yong Ho, Siamak Ebrahimi, Hamidah Mohd Yusof, Huynh Ky, Sheau Wei Tan, Noorjahan Banu Alitheen

**Affiliations:** Institute of Bioscience, Universiti Putra Malaysia, Serdang, Selangor Malaysia; Department of Cell and Molecular Biology, Faculty of Biotechnology and Biomolecular Sciences, Universiti Putra Malaysia, 43400 Serdang, Selangor Malaysia; Department of Bioprocess Technology, Faculty of Biotechnology and Biomolecular Sciences, Universiti Putra Malaysia, 43400 Serdang, Selangor Malaysia; School of Biomedical Sciences, The University of Nottingham Malaysia Campus, Jalan Broga, 43500 Semenyih, Selangor Malaysia; Department of Agriculture Genetics and Breeding, College of Agriculture and Applied Biology, Cantho University, 3/2 Street, Can Tho City, Vietnam

**Keywords:** Anti-inflammation, Chemopreventive, Curry leaves, *Murraya koenigii*, 4T1 cells

## Abstract

**Background:**

The progression of breast cancer is increasing at an alarming rate, particularly in western countries. Meanwhile, the lower incidence in Asian countries could be attributed to the heavy incorporation of green leaves vegetables or spices in their diets. *Murraya koenigii* (MK) or often times known as curry leaves are common spice used mostly in tropical countries. Anti-inflammatory and chemopreventive effects of MK aqueous extract on 4T1 breast cancer cell-challenged mice were evaluated.

**Methods:**

Herein, cytotoxic activity of MK was first tested on 4T1 cells *in vitro*by MTT assay. Then, *in vivo* chemopreventive study was conducted where mice were fed with extracts prior to and after inducing the tumor (inoculation). Tumor size was monitored post-4T1 inoculation. At the end of experiment, histopathology of tumor sections, T cell immunophenotyping, tumor nitric oxide level, serum cytokine level and qPCR analysis on expression of iNOS, iCAM, NF-kB and c-MYC were performed.

**Results:**

MK reduced the tumors’ size and lung metastasis aside from inhibited the viability of 4T1 cells *in vitro*. Furthermore, it decreased the level of nitric oxide and inflammation-related cytokines and genes, including iNOS, iCAM, NF-kB and c-MYC.

**Conclusion:**

The results propose that, MK managed to inhibit the progression of tumor via immunostimulatory effect and inflammatory reaction within the tumor samples. This suggests that MKconsumption could be a savior in the search of new chemopreventive agents.

## Background

Breast cancer is the most common type of cancer among women especiallyin industrialized countries such asEuropean countries and the United States. In other developing countries,for instance, China and India, the relative incidence of breast cancer is much lower and this phenomenon has been related to the different lifestylesin terms of diet and food preparation method [1]. Natural food ingredients commonly consumed in the Asian diet, which carries relatively low toxicity have been proposed as one of the finest chemopreventive strategies to combat cancer [2]. Despite the advance of cancer prevention including better diagnostic for early detection and used of anti-estrogenic drugs such as tamoxifen and raloxifene, which resulted in the reduction of breast cancer incident and mortality, breast cancer is still the most commonly diagnosed cancer that contributes to the second highest caused of cancer associated mortality in women [1, 2]. Cancer chemoprevention has been defined as the use of pharmacologic or natural agents to reverse, suppress, delay or prevent the incident and progression of carcinogenesis [3]. Herbs have been identified as an important source of novel bioactive compounds for medicine development including cancer chemotherapeutic drugs [4]. Lower incident of breast and colon cancer in Asia especially India has been correlated with the diet and natural food ingredients while increasing of breast cancer incident has been correlated with the change of life style including consumption of Western high calorie-dense diet [1].

*Murraya koenigii* (MK) or commonly known as curry leaves is a tropical vegetable that is widely used as spice for seasoning and flavoring in tropical countries. Previous study has shown that MK was good source of nutrient since it contained high amount of insoluble dietary fiber (38.8 %), followed by carbohydrate (12.5 %), crude protein (11.8 %) and crude fat (5.1 %). Besides, MK also contained high amount of potassium (21.5 mg/g), calcium (20.9 mg/g), phosphate (3.6 mg/g), Magnesium (3.1 mg/g) and vitamin C (1.2 mg/g) [5]. Traditionally, MK has been widely used in Ayurveda medicine as tonic, stomachic and carminative due to its medicinal properties [6]. MK has been reportedwith immunomodulatory [7], anti-tumor [7–10], antimicrobial [11], antioxidant [12], anti-inflammatory and analgesic effects [12]. These medicinal benefits maybe contributed by present of the bioactive ingredients in MK. For examples, MK has been reported to contain high amount of polyphenolic, flavonoids, carbazole alkaloids andcoumarins and flavonoids [6]. In terms of flavonoid, myricetin, epicatechin and quercetin were the most common flavonoid compounds present in MK. On the other hand, gallic acid and vanillic acid were the two most common phenolic acids detected in MK [13]. These antioxidant polyphenolics and flavonoids that present in MK contributed to the anti-inflammation, metal-chelating,proteasome inhibitory and apoptotic effects on breast cancer cells [6]. In terms of carbazole alkaloids, MK has been reported to contain Girinimbine [14], mahanine [13, 15], pyrayafoline-D and murrafoline-l [15], which contributed to the *in vitro* cytotoxic effect on liver cancer cell HepG2 [16], acute lymphoblastic T cell MOLT-3, chronic myelogenous leukemia [8], and promyelocytic leukemia cell HL-60 [15]. Antitumor effect via induction of apoptosis and S phase arrest [6] of MK was validated in some *in vivo* modelson Dalton’s ascetic lymphoma [10], dimethyl hydrazine induced colon carcinogenesis [9], and K562 challenged nude mice [8].

Besides antitumor effect, methanolic extract of MK leaves demonstrated a significant immunomodulatory effect where it enhanced the phagocytic index and increased the antibody titer against ovalbumin and protection against cyclophosphamide-induces myelosuppression [7]. Hence, MK holds promise as an immunomodulatory agent acting by stimulating humoral immunity and phagocytic function. Thus, it is interesting to evaluate the potential of MK, one of the major food flavoring ingredients in Indian culture, in preventing incidence of breast cancer. However, it’s potential to prevent breast cancer incident through its antiproliferative, antiinflammatory and immunomodulatory effects were not thoroughly evaluated *in vivo*. We hypothesized that the progression of tumor after inoculation in MK-treated micemay be inhibited, as compared to the untreated in low (LR) and high risk (HR) group mediated at least partially via immunostimulatory effect and inflammatory reaction. Thus, this study was aimed at evaluating the anti-inflammatory, immunomodulatory and chemopreventive effects of MK aqueousextract on 4T1 challenged mice.

## Methods

### Sample collection and extraction

MK was obtained from the curry leaf plantation in Selangor, Malaysia in the months of April to June 2010. The plant was identified and deposited with a voucher number of FRI 65673 by Science Officer Lim Chung Lu from the Forestry Division, Forest Research Institute Malaysia. The leaves were washed, air dried in shade, grounded and extracted with deionized water (ratio 1:8) at 60 °C for 2 h. The extract was filtered with Whatman filter paper no.1 (Millipore,USA) and spray dried at an inlet temperature of 150 °C and outlet temperature of 100 °C (Buchi B-290, Switzerland) (yield 30 % w/w). The extract was stored at −20 °C and was used for the following studies.

### Cell culture

Triple negative mesenchymal-like breast cancer MDA-MB-231 (ATCC HTB-26), murine triple negative (model for stage IV human breast cancer) cell (ATCC CRL-2539) and murine natural killer cell sensitive lymphoma (ATCC TIB-160) (American Type Culture Collection) were maintained in RPMI-1640 medium (Sigma, USA) supplemented with 10 % FBS at 37 °C and 5 % CO_2_.

### *In vitro* MTT cytotoxicity assay

The assay was conducted as follow: 4T1 and MDA-MB-231 cancer cell lines were seeded in 96-well plates at a density of 0.5 × 10^4^cells/well in 100 $\mu$L RPMI. At 24 h after seeding, the medium was removed and the cells were incubated for 3 days with RPMI-1640 in the absence or presence of various concentrations of *M. koenigii* (MK) extracts. The concentration of the extract was 2 fold serial diluted ranging between 5.00 and 0.08 mg/mL. After the incubation period, 20 *μ*L of MTT reagent was added into each well. The plate was incubated again in a CO_2_incubator at 37∘C for 4 h. The resulting MTT-products were determined by measuring the absorbance at 570 nm using an ELISA plate reader (BioTek, USA) [17]. Each point represents the mean of triplicate experiments. The cell viability was determined using the formula below and IC_50_ was calculated based on the graph of viability vs absorbance.

$$ \mathrm{Viability}\left(\%\right) = \frac{\mathrm{optical}\ \mathrm{density}\ \mathrm{of}\ \mathrm{sample}}{\mathrm{optical}\ \mathrm{density}\ \mathrm{of}\ \mathrm{control}}\times 100 $$

### Animals and treatment

For *in vivo* chemopreventive study, 8-week old female BALB/c mice (Total of 42 mice, *n* = 6 per group) were purchased from the Animal house, Universiti Putra Malaysia. The study was approved by the Universiti Putra Malaysia’s Institutional Animal Care and Use Committee (IACUC) (UPM/FPV/PS/3.2.1.551/AUP-R152). The mice were housed in 12 h of light and dark and fed with standard pellet and water *ad libitum*. Mice were oral fed with MK (50 and 200 mg/kg body weight) or normal saline for 30 days. Then, mice from the MK and normal saline fed groups were randomly separated into low (LR) and high (HR) risk cancer groups. MK (50 and 200 mg/kg body weight) and normal saline fed mice were inoculated with 1 × 10^4^ 4T1 cells/mice for LR groups and 1 × 10^6^ 4T1 cells/mice for HR groups on left mammary fat pad subcutaneously, respectively. A group of mice without inoculation served as normal control. MK and normal saline treatment were continued until day 21 post-inoculation. After 21 days of post-inoculation, mice were sacrificed, serum was collected for cytokines (IL1β, IL6, IL10, IL2, IFN-γ) ELISA quantification (BioLegend, USA), spleen for lymphocyte immunophenotyping while tumor was harvested for histopathology and quantitative real time PCR assay.

### Measurement of tumor size

Body weight, tumorssize (with vernier calipers) and tumor incidence were measured twice a week. Tumor volume and tumor burden was calculated using the following formula: tumor volume (mm^3^) = [(width)^2^ × length]/2 and tumor burden (%) = tumor volume (mm^3^)/body weight (mg) × 100 %.

### Histopathalogy study

Lungs were harvested, fixed in 10 % formaldehyde (Sigma-Aldrich, USA) overnight, dried and viewed using microscope to calculate the metastatic tumor nodules on the lung [18]. Primary tumors were dissected and fixed in 10 % formaldehyde, embedded in paraffin, cut into 2-μm sections, and stained with hematoxylin and eosin (H&E) [19].

### Serum cytokine analysis

Serum cytokines were collected from treated and control (untreated) mice after the mammary fat-pad implantation and allowed to clot followed by centrifugation at 3,000xg for 30 min, and immediately frozen and stored at −80 °C until use. Interleukins IL-1β,IL-6, IL-10, IL-2, and Interferon gamma (IFN-γ) levels were analyzed in the sera collected, using the Mouse Inflammation ELISA Strip for Profiling 8 Cytokines (Signosis BioSignal Capture) accordingly.

### Quantitative real- time PCR

RNA extraction of tumor was performed using RNeasy mini plus kit (Qiagen, Germany) and the extracted RNA was converted to cDNA using iScript cDNA synthesis kit (Bio-Rad, USA). Expression of iNOS, NF-kB,iCAM and c-MYC were evaluated using quantitative real time PCR (qRT-PCR) SYBR select master mix (Life Tech, USA) [20].

### Nitric oxide detection

In order to quantify serum and tumor nitric oxide (NO) level, the tumor was collected, homogenized in ice-cold PBS and filtered through 80 μm wire mesh (BD, USA). The filtrates were centrifuged and the supernatant along with previously acquired serum were subjected to nitric oxide quantification using Griess assay (Invitrogen, USA) according to manufacturing protocol.

### Immunophenotyping of spleen cell

Immunophenotyping of the splenocytes isolated from the treated and untreated groups were performed using CD4, CD3 and CD8 antibodies (Abcam, USA) via flow cytometry (BD, USA). The differences between the control or treated group and untreated group were determined by one-way ANOVA.

### *Ex-vivo s*plenocyte cytotoxicity on Yac-1 cell

Effector cells consisted of fresh splenocytes harvested from the mice were co-cultured with Yac-1 (Target) cells and incubated for 18–24 h. The cytotoxicity of splenocytes towards Yac-1 cell line was determined using CytoTox 96 nonradioactive cytotoxicity assay kit (Promega, USA) at the ratio of effector: target of 2: 1 and 10: 1. Assays were performed in triplicate and the percentage of the cytotoxicity was calculated according to the manufacturer protocols.

### Statistical analysis

All tests were carried out with 3 independent experiments and each of the experiment consisted of 3 technical replicates. All results are expressed as Mean ± Standard Error (S.E.M.). Significant levels (*p* < 0.05) were evaluated using ANOVA test (one way) followed by *post hoc* Duncan test.

## Results and discussion

### MK aqueous extract exhibited cytotoxicity on breast cancer cell lines and delay breasttumor incidence in vivo

First of all, we assessed the cytotoxic activity of MK aqueous extracts in human and mouse breast cancer cell lines, MDA-MB231 and 4T1 cells, respectively via *in vitro* MTT cytotoxic assay. MKaqueous extract treatment resulted in time dependent inhibition of MDA-MB-231 (IC_50_ value of 2.40 ± 0.26, 0.80 ± 0.12 and 0.42 ± 0.13 mg/mL) and 4T1 cell viability (IC_50_ value of 1.50 ± 0.90, 0.50 ± 0.11 and 0.37 ± 0.80 mg/mL) for 24, 48 and 72 h of treatment. Overall, MK aqueous extract possessed similar cytotoxicity against both mouse 4T1 cellsand human MDA-MB-231 cells.

**Fig. 1:**
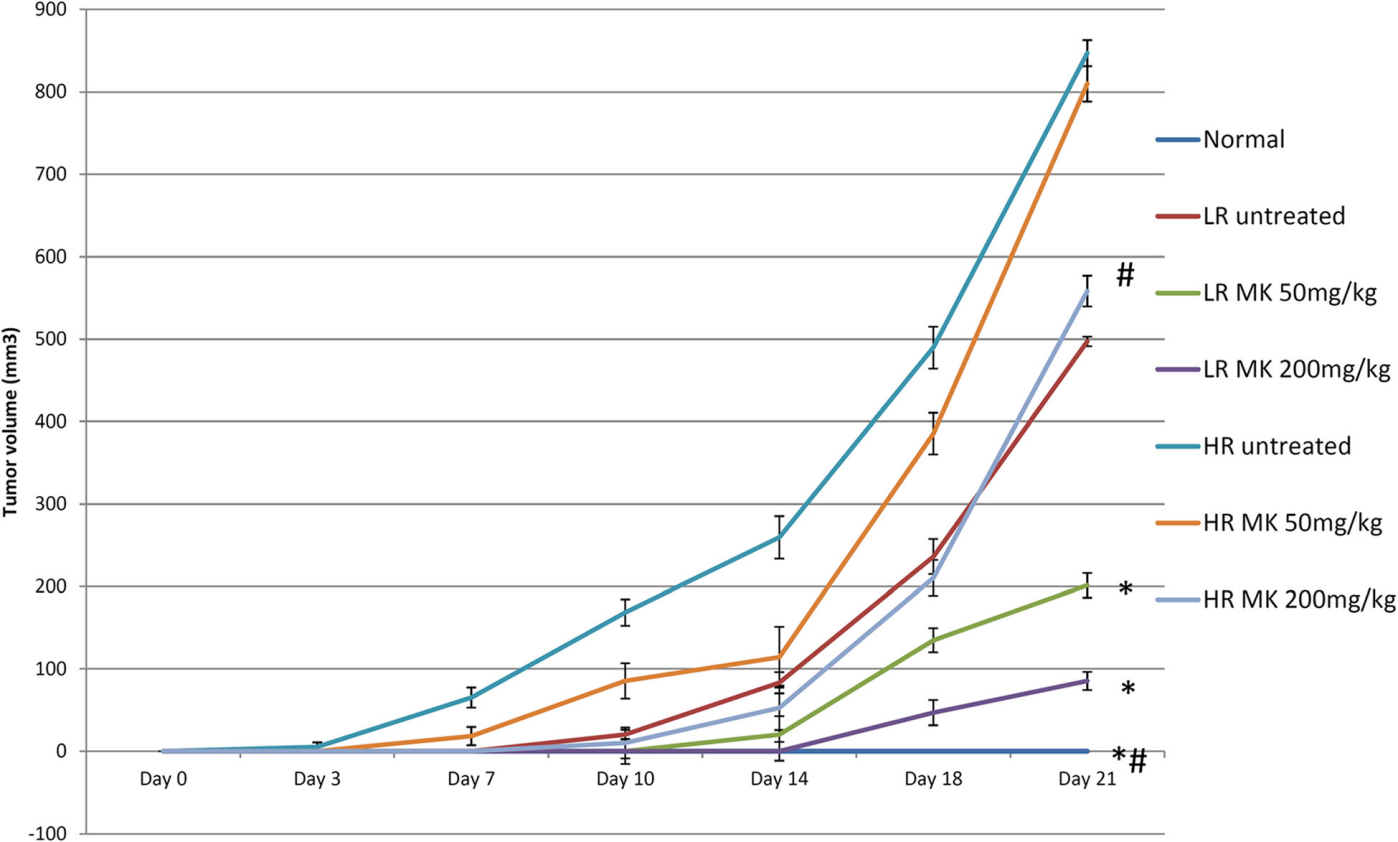
Tumor size: MK delayed the tumor formation in BALB/c mice inoculated s.c. with 4T1 cells in fat pads. The differences between groups were determined by one-way ANOVA (∗for low risk groups while #for high risk groups; *p* < 0.05). Normal: normal healthy mice; LR untreated: untreated low risk mice inoculated with 1 × 10^4^ 4T1 cell/mice; LR MK 50 mg/kg: low risk mice treated with 50 mg/kg of MK extract for 30 days before inoculated with 1 × 10^4^4T1 cell/mice; LR MK 200 mg/kg: low risk mice treated with 200 mg/kg of MK extract for 30 days before inoculated with 1 × 10^4^4T1 cell/mice; HR untreated: untreated high risk mice inoculated with 1 × 10^6^4T1 cell/mice; HR MK 50 mg/kg: high risk mice treated with 50 mg/kg of MK extract for 30 days before inoculated with 1 × 10^6^4T1 cell/mice; HR MK 200 mg/kg: high risk mice treated with 200 mg/kg of MK extract for 30 days before inoculated with 1 × 10^6^4T1 cell/mice inoculated mice. LR: Low risk groups. HR: High risk groups

**Fig. 2:**
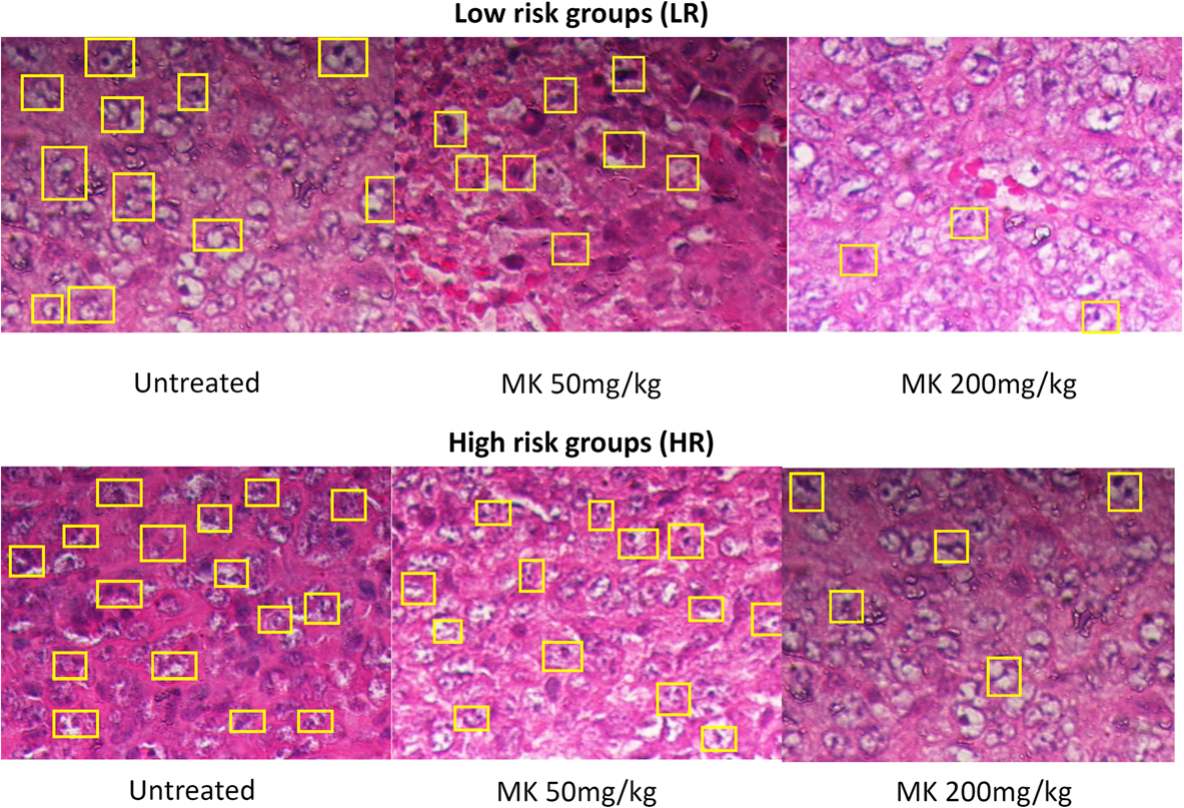
Histological study: Histological emergence of the 4T1 tumor from LR and HR groups. Boxes indicate cells under mitotic division. Black bars signify 200 *μ*m (magnification 40x). The differences between the control or treated group and untreated group were determined by one-way ANOVA (∗for low risk groups while #for high risk groups; *p* < 0.05). Normal: normal healthy mice; LR untreated: untreated low risk mice inoculated with 1 × 10^4^4T1 cell/mice; LR MK 50 mg/kg: low risk mice treated with 50 mg/kg of MK extract for 30 days before inoculated with 1 × 10^4^ 4T1 cell/mice; LR MK 200 mg/kg: low risk mice treated with 200 mg/kg of MK extract for 30 days before inoculated with 1 × 10^4^4T1 cell/mice; HR untreated: untreated high risk mice inoculated with 1 × 10^6^4T1 cell/mice; HR MK 50 mg/kg: high risk mice treated with 50 mg/kg of MK extract for 30 days before inoculated with 1 × 10^6^4T1 cell/mice; HR MK 200 mg/kg: high risk mice treated with 200 mg/kg of MK extract for 30 days before inoculated with 1 × 10^6^4T1 cell/mice inoculated mice

Similar to our *in vitro* data, many studies have reported the *in vitro* [6, 8, 16] and*in vivo* [8, 10, 21] antitumor effect of MK [6]. However, the *in vivo* chemopreventive and anti-tumor immunomodulatory effects were not well evaluated. In thisstudy, *in vivo* pre-treatment with MKaqueous extract in 4T1 challenged mice resulted in delay tumor development (Table 1) with small tumor size (Fig. 1) and lower tumor burden (Table 1) in dosage dependent manner in both LR and HR groups (Fig. 1). Although development of tumor was observed in all 4T1 challenged mice, high concentration of MK aqueous extract demonstrated the highest prevention where it delayed the formation of tumor comparing to untreated 4T1 challenged mice in both LR and HR groups. Previous findings reported that MK methanol extract effectively arrest the cell division at S phase along with apoptosis inductionon MDA-MB-231 cell. Our histological analysis revealedthat mitoses were frequently observed in the tumors of the untreated HR and LR groups of mice (Fig. 2). Treatment with MK was able to reduce mitotic division in the tumors of the LR group of mice in Fig. 2. We also observed the lung harvested from untreated LR and HR mice were recorded with ~23 and ~15 metastatic nodules. 200 mg/kg bw of MK aqueous extract was recorded with significantly (*p* < 0.05) lower metastatic nodules, which were ~4 and ~14 in the lung of LR and HR mice, respectively (Table 2). Overall, high dose of MK aqueous extract was able to reduce incidence of cell underwent mitotic division in both LR and HR mice.

**Table 1:** Tumor incidence and tumor burden at day 21 post 4T1 injection

Groups	Day 3		Day 7		Day 10		Day 14		Day 18		Day 21	
	TI	TB (%)	TI	TB (%)	TI	TB (%)	TI	TB (%)	TI	TB (%)	TI	TB (%)
Normal	-	-	-	-	-	-	-	-	-	-	-	-
LR untreated	0/6	0.00	0/6	0.00	4/6	0.10	6/6	0.42	6/6	1.00	6/6	2.11
LR MK 50 mg/kg	0/6	0.00	0/6	0.00	0/6	0.00	5/6	0.10	6/6	0.55	6/6	0.82
LR MK 200 mg/kg	0/6	0.00	0/6	0.00	0/6	0.00	0/6	0.00	5/6	0.20	6/6	0.36
HR untreated	4/6	0.03	6/6	0.33	6/6	0.84	6/6	1.30	6/6	2.11	6/6	3.65
HR MK 50 mg/kg	0/6	0.00	4/6	0.00	6/6	0.43	6/6	0.57	6/6	1.72	6/6	3.61
HR MK 200 mg/kg	0/6	0.00	0/6	0.00	4/6	0.05	5/6	0.26	6/6	0.84	6/6	2.23

TI: tumor incidence (tumor bearing mice/total mice per group); TB: tumor burden

**Table 2:** Lung metastasis at day 21 post 4T1 injection

Groups	Number of metastatic lung nodules/mouse
Normal	-^*,#^
LR untreated	15 ± 4
LR MK 50 mg/kg	7 ± 3^*^
LR MK 200 mg/kg	4 ± 2^*^
HR untreated	23 ± 6
HR MK 50 mg/kg	19 ± 5
HR MK 200 mg/kg	16 ± 3^#^

Note: Values are mean ± SEM from triplicate analyses. Data are significantly different from LR untreated (*p < 0.05) and HR untreated (^#^ *p* < 0.05) by ANOVA and followed by Duncan test

### MK Aqueous extracts possess immmunomodulatary and anti-inflammation properties by regulating several immune-related players

**Fig. 3:**
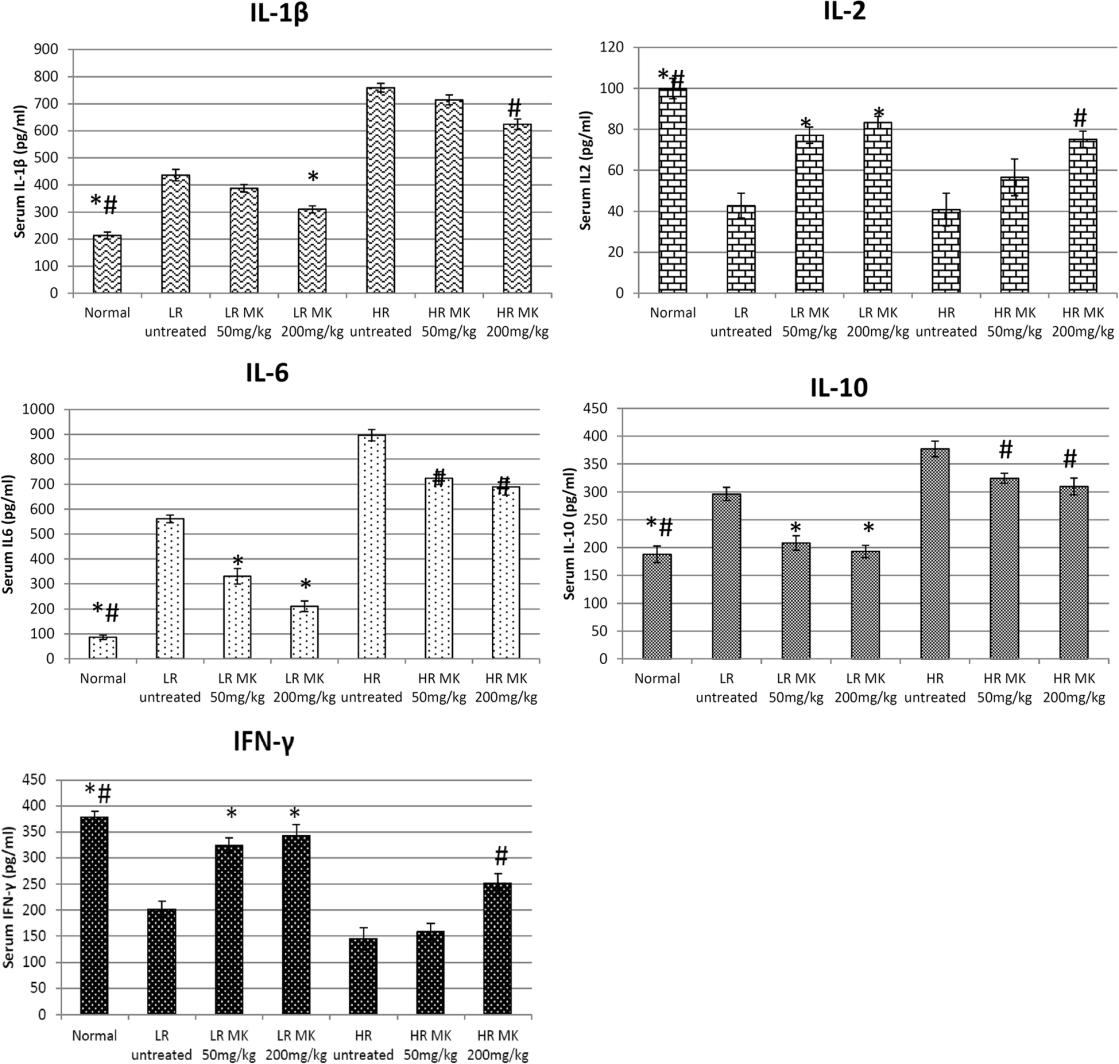
Quantitative real time PCR: Quantitative real timePCR evaluation on expression of tumor iNOS, NF-kB, c-MYC and iCAM of MK (50 and 200 mg/kg) treated and untreated mice. The statistical differences among all groups were assessed by one-way ANOVA followed by Duncan post-hoc. The differences between the control or treated group and untreated group were determined by one-way ANOVA (∗*p* < 0.05). Normal: normal healthy mice; LR untreated: untreated low risk mice inoculated with 1 × 10^4^4T1 cell/mice; LR MK 50 mg/kg: low risk mice treated with 50 mg/kg of MK extract for 30 days before inoculated with 1 × 10^4^4T1 cell/mice; LR MK 200 mg/kg: low risk mice treated with 200 mg/kg of MK extract for 30 days before inoculated with 1 × 10^4^4T1 cell/mice; HR untreated: untreated high risk mice inoculated with 1 × 10^6^4T1 cell/mice; HR MK 50 mg/kg: high risk mice treated with 50 mg/kg of MK extract for 30 days before inoculated with 1 × 10^6^4T1 cell/mice; HR MK 200 mg/kg: high risk mice treated with 200 mg/kg of MK extract for 30 days before inoculated with 1 × 10^6^4T1 cell/mice inoculated mice. LR: Low risk groups. HR: High risk groups

**Fig. 4:**
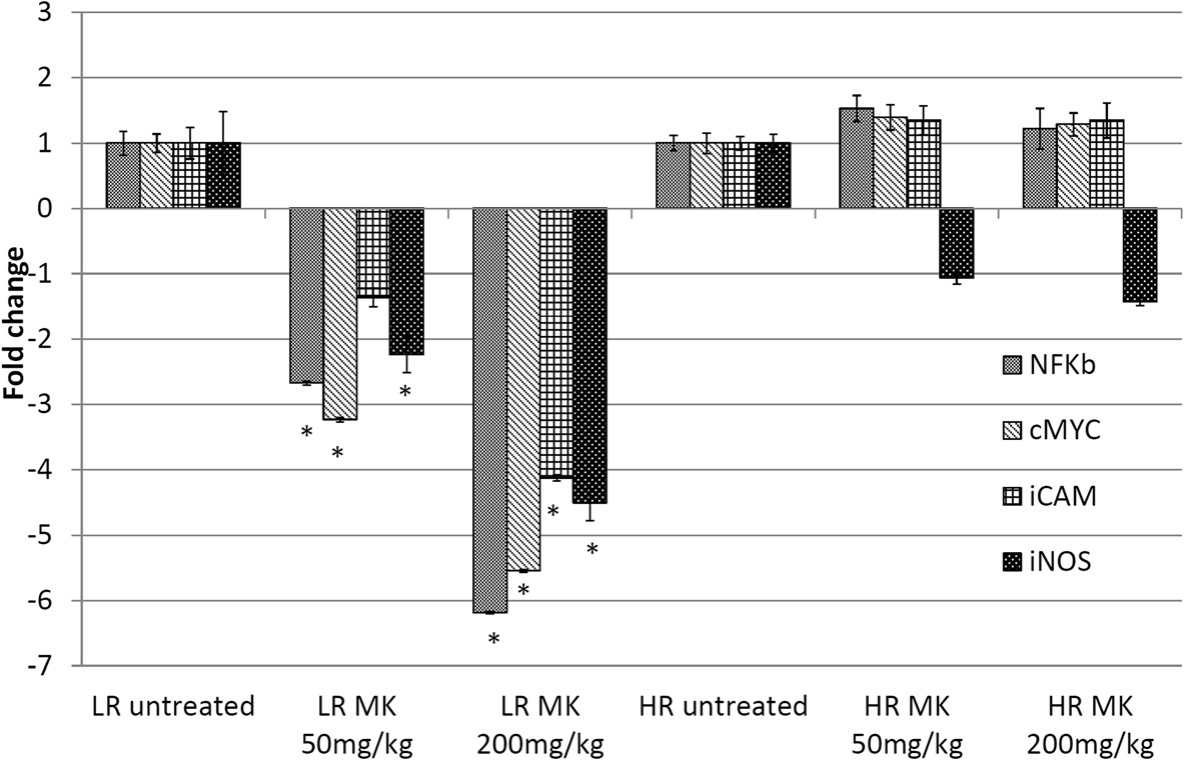
Serum levels ofIL1β, IL6, IL10, IL2, IFN-γ (pg/mL) from different treatment groups on day 21 after 4T1 cell inoculation. Each value represents the means ± S.D. for three mice in triplicate each. The differences between the control or treated group and untreated group were determined by one-way ANOVA (∗for low risk groups while #for high risk groups; *p* < 0.05). Normal: normal healthy mice; LR untreated: untreated low risk mice inoculated with 1 × 10^4^4T1 cell/mice; LR MK 50 mg/kg: low risk mice treated with 50 mg/kg of MK extract for 30 days before inoculated with 1 × 10^4^4T1 cell/mice; LR MK 200 mg/kg: low risk mice treated with 200 mg/kg of MK extract for 30 days before inoculated with 1 × 10^4^4T1 cell/mice; HR untreated: untreated high risk mice inoculated with 1 × 10^6^4T1 cell/mice; HR MK 50 mg/kg: high risk mice treated with 50 mg/kg of MK extract for 30 days before inoculated with 1 × 10^6^4T1 cell/mice; HR MK 200 mg/kg: high risk mice treated with 200 mg/kg of MK extract for 30 days before inoculated with 1 × 10^6^4T1 cell/mice inoculated mice. LR: Low risk groups. HR: High risk groups

**Fig. 5:**
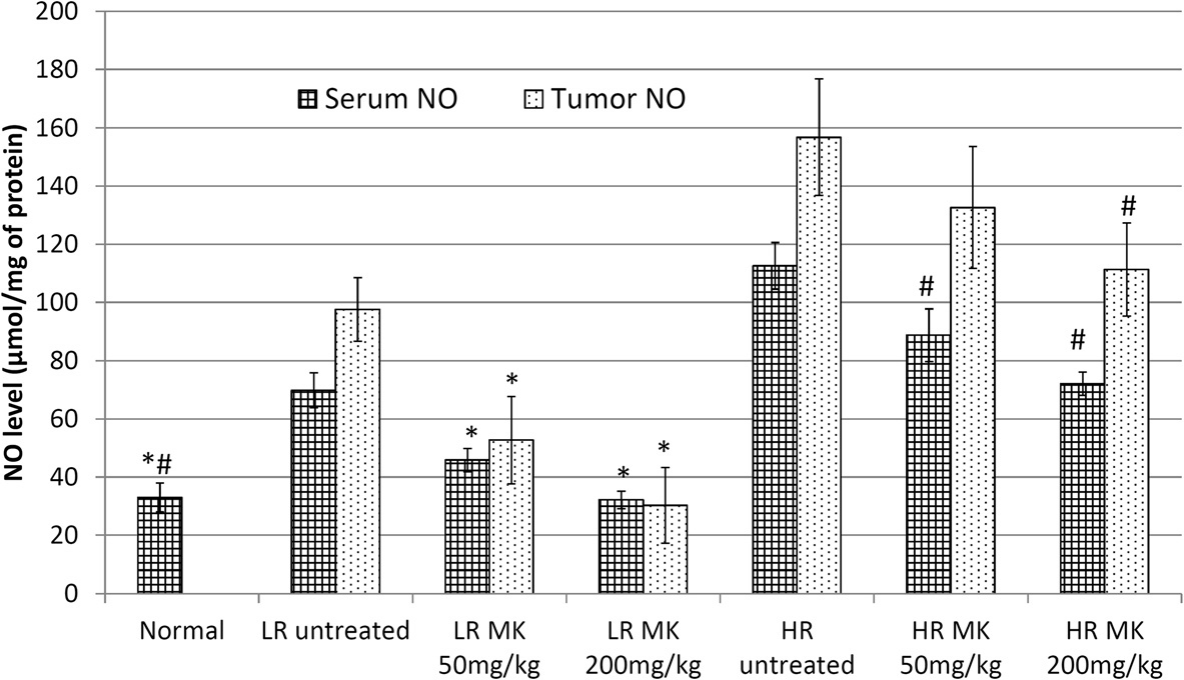
NO level: NO level of tumor and spleen homogenate from different treatment groups on day 21 following 4T1 cell inoculation. Each value represents the means ± S.D. for three mice in triplicate each. The differences between the control or treated group and untreated group were determined by one-way ANOVA (∗for low risk groups while #for high risk groups; *p* < 0.05). Normal: normal healthy mice; LR untreated: untreated low risk mice inoculated with 1 × 10^4^4T1 cell/mice; LR MK 50 mg/kg: low risk mice treated with 50 mg/kg of MK extract for 30 days before inoculated with 1 × 10^4^4T1 cell/mice; LR MK 200 mg/kg: low risk mice treated with 200 mg/kg of MK extract for 30 days before inoculated with 1 × 10^4^4T1 cell/mice; HR untreated: untreated high risk mice inoculated with 1 × 10^6^4T1 cell/mice; HR MK 50 mg/kg: high risk mice treated with 50 mg/kg of MK extract for 30 days before inoculated with 1 × 10^6^4T1 cell/mice; HR MK 200 mg/kg: high risk mice treated with 200 mg/kg of MK extract for 30 days before inoculated with 1 × 10^6^4T1 cell/mice inoculated mice. LR: Low risk groups. HR: High risk groups

Apart from antitumor effect, the immunomodulatory effect of MKon humoral and macrophage activation has been previously evaluated [13]. However, study on the activation of immune system to prevent tumor development or progression are lacking. Phytochemicals such as green tea catechin, garlic organosulfur and curcumin that possessed anti-inflammatory and immunostimulatory effect have been demonstrated as good chemopreventive effect [21, 22]. Chronic inflammation and attenuation of antitumor immunity including inhibition of cytotoxic T lymophocyte and natural killer cell cytotoxicity were found to support tumor outgrowth [23]. In this study, NF-kB, iNOS, NO, IL-1β, IL-10 and IL-6 were evaluated to monitor the inflammation in the untreated and MK treated LR and HR mice. Overexpression of NF-kB that regulates the inflammationhas been found to promote cancer anti-apoptosis, angiogenesis and metastasis [17]. High dose of MKaqueous extract had the highest reduction of the NF-kB in LR group (Fig. 3). NF-kB can be activated by proinflammatory cytokineIL-1β and pleiotropic cytokine IL-6. IL-6 stimulated breast cancer cell proliferation, anti-apoptotic effect, motility and invasion thus promote progression of breast cancer [24]. On the other hand, the immunosuppressive cytokine IL-10 has been reported with both pro and anti-tumor effect [25]. Generally, elevated level of serum IL-6 and IL-10 were found to correlate strongly with the breast cancer clinical stage especially in the metastatic estrogen receptor negative breast cancer [26, 27]. Elevated level of serum IL-1β, IL-6 and IL-10 were observed in untreated LR and HR mice comparing to normal control. Our findings show that the inflammatory mediator IL-6, proinflammatory cytokine IL-1β and immunosuppressive cytokine IL-10 in serum levels were reduced in MK-treated 4T1 inoculated mice especially in the LR group (Fig. 4). Subsequently, high dose of MKaqueous extract had the highest reduction of iNOS in HR group (Fig. 3). iNOS catalyzes oxidative deamination of l-arginine to produce the pro-inflammatory mediator NO. Raised of NO involved in promotion of extreme oxidation, tumor progression and angiogenesis [28]. The presence of inflammation in the serum and tumor was measured by NO quantification. Figure 5 showed that the progression of cancer was linked with higher levels of NO in both serum and tumor. MK-treatments successfully reduced the NO levels in both the spleen and tumor of both the LR and HR groups of tumor. Previous reporthas shown that methanol extract of MK possessed anti-inflammatory effect [12]. From our current study, it indicates that MKaqueous extract effectively reduced inflammation mediators and cytokines, which help to delay the progression of breast cancer especially in the LR mice. Metastatic nodules reduction in the lung harvested from MK aqueous extract mice in both LR and HR groups further confirmed it. The metastasis incident was further supported by the expression of iCAM in tumor, which was evaluated by quantitative real time PCR. iCAM is an adhesion molecule in the immunoglobulin superfamily correlated with metastatic potential where its over-expression promotes transendothelial migration of the cancer cell [29]. iCAM expression was significantly downregulated in the tumor of MK*-*treated LR mice (Fig. 3), which contributed to the control of primary tumor progression and metastasis of tumor by the extract.

Overexpression of c-MYC was found to drive cellular proliferation thus promote tumorigenesis whereas downregulation of c-MYC can help to restore cellular senescence and thus promote tumor regression [30]. In line with our finding, c-MYC expression was found to be effectively downregulated in MKaqueous extract treated LR mice (Fig. 3). This study proposes that delay of 4T1 cancer progression in LR mice by MK aqueous extract might be contributed by the cell cycle arrest in the dosage dependent manner as supported by the histology analysis and c-MYC expression. Nevertheless, other factors could add to its chemopreventive effect since the c-MYC expression in HR group was not much different than untreated mice.

**Fig. 6:**
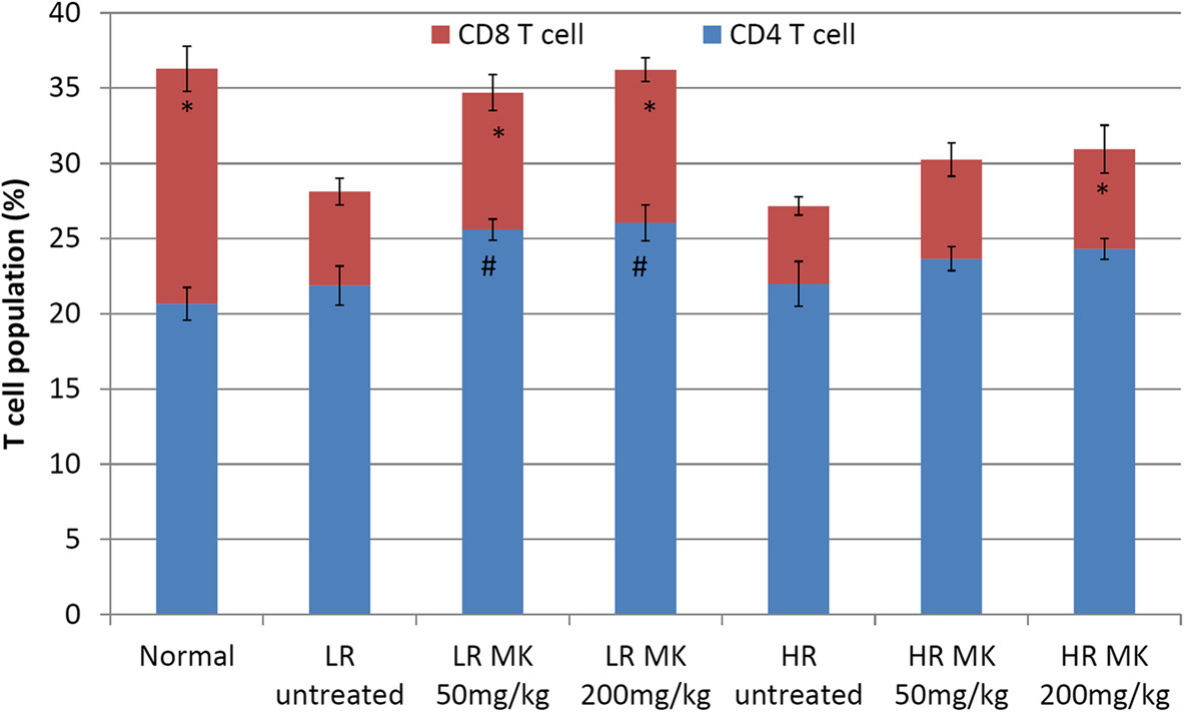
Immunophenotyping: CD4 and CD8 immunophenotyping of samples from different treatment groups on day 21 after 4T1 cell inoculation. Each value represents the means ± S.D. for three mice in triplicate each. The differences between the control or treated group and untreated group were determined by one-way ANOVA (∗for low risk groups while #for high risk groups; *p* < 0.05). Normal: normal healthy mice; LR untreated: untreated low risk mice inoculated with 1 × 10^4^4T1 cell/mice; LR MK 50 mg/kg: low risk mice treated with 50 mg/kg of MK extract for 30 days before inoculated with 1 × 10^4^4T1 cell/mice; LR MK 200 mg/kg: low risk mice treated with 200 mg/kg of MK extract for 30 days before inoculated with 1 × 10^4^4T1 cell/mice; HR untreated: untreated high risk mice inoculated with 1 × 10^6^4T1 cell/mice; HR MK 50 mg/kg: high risk mice treated with 50 mg/kg of MK extract for 30 days before inoculated with 1 × 10^6^4T1 cell/mice; HR MK 200 mg/kg: high risk mice treated with 200 mg/kg of MK extract for 30 days before inoculated with 1 × 10^6^4T1 cell/mice inoculated mice. LR: Low risk groups. HR: High risk groups

**Fig. 7:**
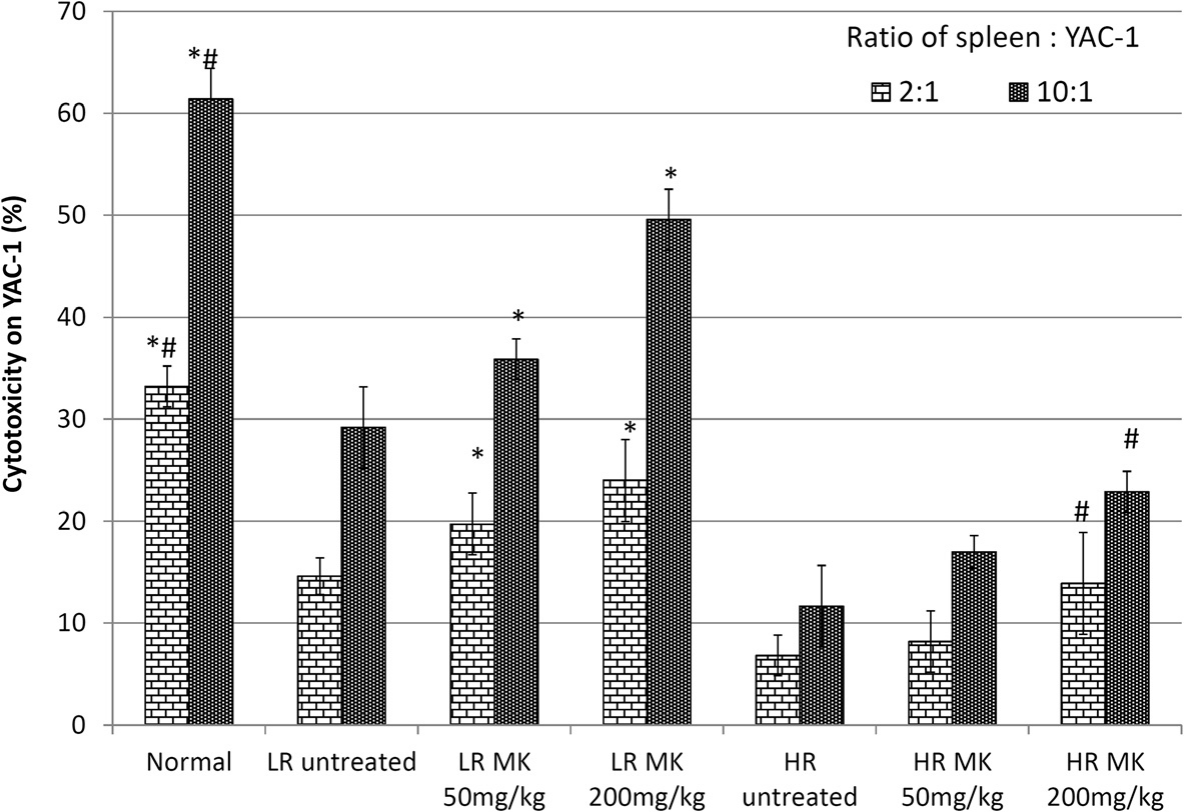
Cocultivation Cytotoxicity: Cytotoxicity level of splenocyte on Yac-1 at E: T ratio of 2 : 1 and 10 : 1 from different treatment groups on day 21 after 4T1 cell inoculation. Each value represents the means ± S.D. for three mice in triplicate each. The differences between the control or treated group and untreated group were determined by one-way ANOVA (∗for low risk groups while #for high risk groups; *p* < 0.05). Normal: normal healthy mice; LR untreated: untreated low risk mice inoculated with 1 × 10^4^4T1 cell/mice; LR MK 50 mg/kg: low risk mice treated with 50 mg/kg of MK extract for 30 days before inoculated with 1 × 10^4^4T1 cell/mice; LR MK 200 mg/kg: low risk mice treated with 200 mg/kg of MK extract for 30 days before inoculated with 1 × 10^4^4T1 cell/mice; HR untreated: untreated high risk mice inoculated with 1 × 10^6^4T1 cell/mice; HR MK 50 mg/kg: high risk mice treated with 50 mg/kg of MK extract for 30 days before inoculated with 1 × 10^6^4T1 cell/mice; HR MK 200 mg/kg: high risk mice treated with 200 mg/kg of MK extract for 30 days before inoculated with 1 × 10^6^4T1 cell/mice inoculated mice. LR: Low risk groups. HR: High risk groups

Additionally, MK was also able to restore the production of both IL-2 and IFN-*γ* in the LR group of mice and high dose of MK in HR group, whereas no significant changes at low dose of HR group (Fig. 4). IL-2 is the T helper 1 (Th1) cytokines that promote CD8 CTL proliferation and cytolytic activity against cancer. On the other hand, type II interferon IFN-γ produced by activated CD8 CTL to inhibit angiogenesis, promote CTL and NK cell cytotoxicity, positively feedback on the Th1 immunity while suppress Th2 cell differentiation and promote tumor eradication via apoptosis [23]. The treatment of MK *in vivo* managed to increase the level of both CD8 CTLs and CD4 T cells in both the HR and LR groups (Fig. 6). Increment of *ex-vivo* cytotoxicity against 4T1 cells indicates the improvement of antitumor immunity. Co-cultivation of splenocyte with Yac-1 cell was used to evaluate the cytotoxicity of the splenocyte on day 21 following inoculation of 4T1 cell. A higher ratio of effector (splenocytes) was associated with greater cytotoxicity against 4T1 cells. However, this effect was significantly (*P*< 0.05) lower in the untreated mice. Treatments with MK was able to enhance splenocyte cytotoxicity against 4T1 cells in LR mice while in HR group, treatment with high dose of MK was able to slightly enhance the splenocyte cytotoxicity as compared to the untreated group of mice as shown in Fig. 7. In general, higher level of antitumor serum cytokines (IL-2 and IFN-γ) (Fig. 4) associated with improved splenocyte cytotoxicity against 4T1 cells (Fig. 7) were observed in MK*-*treated mice in dosage dependent manner. Better recovery on the antitumor immunity was observed in LR mice than the HR mice treated with the extract. This signifies that chemoprevention of high dose of extract in HR mice maybe contributed by its immunostimulatory effect. On the other hand, the extract effectively controls the progression of 4T1 in LR mice by its antiproliferation, antiinflammation and immunomodulation effects.

## Conclusion

MKaqueous extract had demonstrated chemopreventive effects on 4T1 challenged mice contributed by the cytotoxicity, anti-inflammatory and immunomodulatory effects. Moreover, MK aqueous extract has delayed the formation of breast cancer and reduced the mitotic division of the tumor through stimulation of T cell cytokine production (IL-2 and IFN-*γ*) and cytotoxicity. Here we show that MKaqueous extract possessed potential chemopreventive effect especially on the LR of breast cancer.

## Abbreviations

MK: *Murrayakoenigii*
4T1: Murine breast cancer cell
LR: Low riskcancer
HR: High risk cancer
CTL: Cytolytic T lymphocyte
NK: Natural killer
IFN-*γ*: Interferon gamma
IL: Interleukin
NO: Nitric oxide
iNOS: inducible nitric oxide synthase
